# Initial Experience With Endoscopic Ultrasound‐Guided Gallbladder Drainage Using Lumen‐apposing Metal Stents in Extremely Elderly and Frail Patients

**DOI:** 10.1002/deo2.70369

**Published:** 2026-06-22

**Authors:** Kiyoyuki Kobayashi, Takako Nomura, Maki Ayaki, Tomohiro Ogi, Yudai Sato, Manabu Yamada, Daisuke Namima, Naoki Fujita, Hiroki Yamana, Hideki Kamada, Toshiharu Funaki, Akihiro Kondo, Yasuhisa Ando, Hironobu Suto, Minoru Oshima, Keiichi Okano, Hideki Kobara

**Affiliations:** ^1^ Department of Gastroenterology and Hepatology HITO Medical Center Shikokuchuo Japan; ^2^ Division of Innovative Medicine for Hepatobiliary and Pancreatology Faculty of Medicine Kagawa University Kita‐gun Japan; ^3^ Department of Gastroenterology and Neurology Faculty of Medicine Kagawa University Kita‐gun Japan; ^4^ Department of Gastroenterology Kaisei Hospital Sakaide Japan; ^5^ Department of Gastroenterological Surgery, Faculty of Medicine Kagawa University Kita‐gun Japan

**Keywords:** acute cholecystitis, endoscopic ultrasound, frailty, gallbladder, stents

## Abstract

**Objectives:**

Endoscopic ultrasound‐guided gallbladder drainage (EUS‐GBD) using lumen‐apposing metal stents (LAMS) is a treatment option for acute cholecystitis in high‐risk surgical patients. However, data on extremely elderly and frail patients are limited. We report our initial experience with EUS‐GBD using electrocautery‐enhanced LAMS in this population after reimbursement approval in Japan.

**Methods:**

We retrospectively analyzed consecutive patients who underwent EUS‐GBD using electrocautery‐enhanced LAMS for acute cholecystitis at a single center after reimbursement approval in June 2025.

**Results:**

Ten patients (median age, 90 years; median Clinical Frailty Scale score, 7) underwent EUS‐GBD. All procedures were performed via a transduodenal approach using a 10‐mm LAMS. Technical success was achieved in all patients. Clinical success was achieved in nine of the 10 patients (90%); one patient with Grade III cholecystitis complicated by severe pneumonia and heart failure died on postoperative day 1 before clinical assessment. The median stent deployment time was 3.5 min, and the median total procedure time was 16 min. Cholecystoscopy through the LAMS using a slim endoscope was feasible when required.

**Conclusion:**

In this pilot study, EUS‐GBD using electrocautery‐enhanced LAMS was technically successful in extremely elderly and frail patients. Although short‐term outcomes appeared acceptable in most patients, the single POD1 death underscores the importance of careful patient selection. These preliminary findings require validation in larger studies.

**Trial Registration:**

N/A.

## 1 Introduction

Acute cholecystitis requires prompt intervention. Although laparoscopic cholecystectomy is the standard treatment, many patients are poor surgical candidates because of advanced age, frailty, and comorbidities [1, 2]. Gallbladder drainage is recommended as an alternative to surgery in these patients [2].

Gallbladder drainage includes percutaneous transhepatic gallbladder drainage (PTGBD), endoscopic transpapillary GBD (ETGBD), and endoscopic ultrasound‐guided GBD (EUS‐GBD) [2]. PTGBD is the standard nonsurgical modality, but it is associated with tube discomfort, accidental dislodgement, repeated interventions, and recurrent cholecystitis after tube removal [3, 4]. ETGBD provides internal drainage without an external tube; however, its success is limited by cystic duct tortuosity, stones, endoscopic retrograde cholangiopancreatography (ERCP)‐related risks, including post‐ERCP pancreatitis [5].

EUS‐GBD has emerged as an alternative procedure. Lumen‐apposing metal stents (LAMS), particularly electrocautery‐enhanced devices, enable single‐step stent deployment [6, 7]. In high‐risk patients, the European Society of Gastrointestinal Endoscopy guidelines recommend EUS‐GBD over PTGBD owing to fewer adverse events and reinterventions [8]. However, most studies have involved relatively young patients, and data on EUS‐GBD in extremely elderly and frail patients remain limited. Moreover, frailty assessment has rarely been incorporated into EUS‐GBD studies despite its established importance in elderly patients undergoing invasive procedures.

In Japan, the Hot AXIOS system (Boston Scientific, Marlborough, MA, USA) is currently the only LAMS approved for EUS‐GBD. Reimbursement was approved in June 2025 with appropriate use guidelines established by the Japan Gastroenterological Endoscopy Society and the Japan Biliary Association [9]. This has expanded the availability of EUS‐GBD; however, Japanese clinical data remain scarce. This issue is particularly relevant in Japan's super‐aged society, where the number of extremely elderly and frail patients with acute cholecystitis is increasing, and minimally invasive internal drainage may offer practical advantages.

Therefore, this study reports our initial experience and clinical outcomes of EUS‐GBD using electrocautery‐enhanced LAMS in extremely elderly and frail patients during the early post‐reimbursement period in Japan.

## 2 Methods

### 2.1 Study Design and Patients

This single‐center retrospective study included consecutive patients who underwent EUS‐GBD using the Hot AXIOS system for acute cholecystitis (September 2025–February 2026) at HITO Medical Center. Patients were considered for EUS‐GBD if they met the following criteria based on the Japanese appropriate use guidelines: (1) diagnosis of acute cholecystitis according to the Tokyo Guidelines 2018 [1]; (2) high surgical risk [Charlson Comorbidity Index (CCI) ≥6 and/or American Society of Anesthesiologists Physical Status (ASA‐PS) score ≥3]; and (3) multidisciplinary agreement that cholecystectomy was inappropriate. In patients for whom PTGBD or ETGBD was considered, the final drainage strategy was based on anatomical feasibility, disease severity, and endoscopic/radiologic assessment. Patients with suspected gangrenous or perforated cholecystitis were excluded because LAMS use for these conditions is not permitted under the Japanese guidelines. The study was approved by the institutional review board of HITO Medical Center (approval number: [20260306001]), and informed consent was obtained from all patients or their legal representatives.

### 2.2 EUS‐GBD Procedure

All procedures were performed by a single endoscopist certified for Hot AXIOS use. A 10‐mm LAMS was used at our institution. Procedures were performed under intravenous propofol sedation with supplemental oxygen (2 L/min via nasal cannula) and continuous cardiopulmonary monitoring, including pulse oximetry, electrocardiography, blood pressure measurement, and bispectral index monitoring. Antibiotics were administered intravenously before the procedure. A linear echoendoscope (GF‐UCT260; Olympus, Tokyo, Japan) was used to identify the gallbladder. After confirming an avascular puncture path using color Doppler, the gallbladder was punctured with a 10‐mm electrocautery‐enhanced LAMS (Hot AXIOS System; Boston Scientific, Marlborough, MA, USA). The distal flange was deployed within the gallbladder under EUS guidance, and the proximal flange was deployed in the duodenal lumen under direct endoscopic visualization. Bile and/or pus drainage was confirmed, and the procedure was completed. Fluoroscopy was used in all cases to confirm the echoendoscope position before puncture and appropriate stent placement after LAMS deployment. At our institution, the transduodenal approach from the duodenal bulb is used when a safe puncture tract is confirmed.

### 2.3 Post‐procedural Management

The patients were monitored for symptom resolution and improvement in inflammatory markers. Follow‐up information was obtained through inpatient observations, outpatient visits, and telephone interviews. If endoscopy was considered safe and the patient and family desired further intervention, follow‐up cholecystoscopy was performed using a slim upper endoscope (GIF‐1200N; Olympus, Tokyo, Japan; outer diameter, 5.4 mm) through a 10‐mm LAMS for gallstone assessment and removal. Subsequent cholecystoscopy with stone removal was considered when the patient could communicate, the patient and family desired further intervention, and the overall clinical condition was suitable. For long‐term stent management, because the long‐term outcomes of permanent LAMS placement remain uncertain, elective replacement with a plastic stent was offered after discussion with the patient and family.

### 2.4 Definitions and Outcomes

Technical success was defined as successful LAMS deployment with adequate apposition confirmed using EUS and/or fluoroscopy. Clinical success was defined as resolution of symptoms (abdominal pain and fever) within 72 h and improvement in inflammatory markers (white blood cell count [WBC] and C‐reactive protein [CRP]) 48–72 h post‐procedure, without additional drainage. Adverse events were classified according to the ASGE lexicon [10]. Frailty was assessed using the Clinical Frailty Scale (CFS) [11]. Procedure time was defined as the interval from gallbladder puncture to stent deployment. The total procedure time was defined as the interval from endoscope insertion to withdrawal, including EUS observation and puncture‐site selection. The follow‐up duration was calculated from the procedure date to the last contact or death.

### 2.5 Statistical Analysis

Continuous variables are expressed as median (range) and categorical variables as number (percentage). Changes in inflammatory markers (WBC and CRP) between admission and 48–72 h post‐procedure were compared using the Wilcoxon signed‐rank test. Statistical significance was set at *p* < 0.05. Statistical analyses were performed using EZR (Jichi Medical University, Tochigi, Japan), a graphical user interface for R (R Foundation for Statistical Computing, Vienna, Austria) [12].

## 3 Results

### 3.1 Patient Characteristics

**FIGURE 1:**
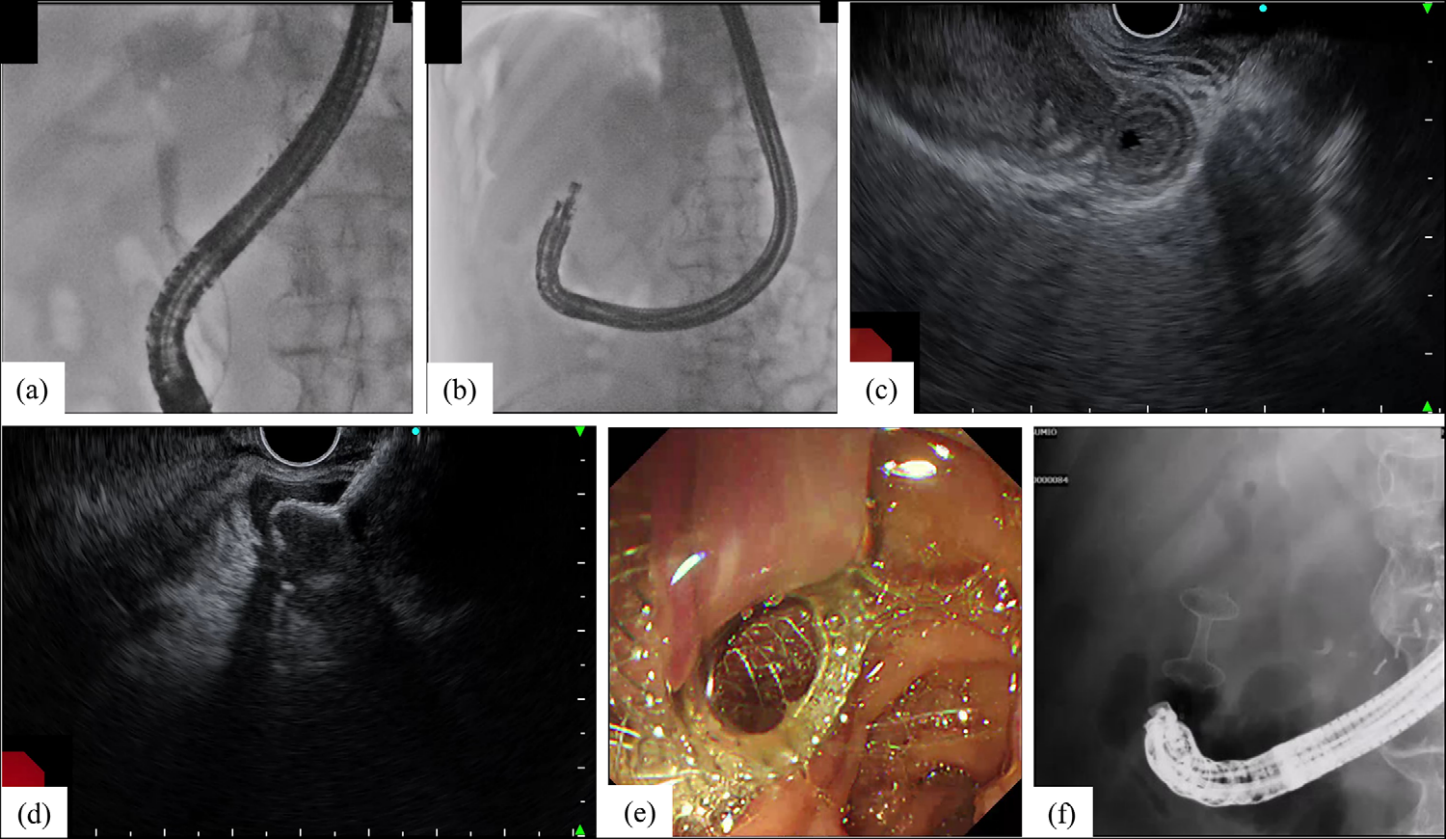
Rescue endoscopic ultrasound (EUS)‐guided gallbladder drainage in a patient with Billroth‐I reconstruction after failed endoscopic transpapillary gallbladder drainage. (a) Fluoroscopic image showing attempted cystic duct cannulation during endoscopic retrograde cholangiopancreatography; the cystic duct branched downward and was suspected to have been narrowed due to inflammation, preventing guidewire advancement into the gallbladder. (b) Fluoroscopic image demonstrating echoendoscope positioning in a patient with Billroth‐I reconstruction. (c) Endoscopic ultrasound image showing a distended gallbladder with stones. (d) Deployment of the distal flange of the 10‐mm lumen‐apposing metal stent within the gallbladder under EUS guidance. (e) Endoscopic view of the deployed lumen‐apposing metal stent with drainage of infected bile. (f) Fluoroscopic image confirming appropriate stent positioning.

Ten patients underwent EUS‐GBD. Tables 1 and 2 present the patient characteristics and individual case details, respectively. Median age was 90 years (range, 77–98 years; female, *n* = 6). All patients had substantial comorbidities, with a median CCI of 8 (range, 7–9) and a median ASA‐PS of 3 (range, 2–4). All patients were frail, with a median CFS of 7 (range, 5–7), indicating moderate to severe frailty. Five (50%) patients had active malignancy. Common reasons for surgical ineligibility included advanced age, bedridden status, communication difficulties, and comorbidities. According to the Tokyo Guidelines 2018, four patients (40%) had Grade I, four (40%) Grade II, and two (20%) Grade III acute cholecystitis. One patient (Patient 9) had a prior distal gastrectomy with Billroth‐I reconstruction. ETGBD failed because of difficult cystic duct cannulation. EUS‐GBD was successfully performed as a rescue procedure (Figure 1).

**TABLE 1:** Patient characteristics (*n* = 10).

Variable	Value
Age, years	90 (77–98)
Female sex	6 (60)
BMI, kg/m^2^	19.0 (14.8–26.8)
Charlson Comorbidity Index	8 (7–9)
ASA‐PS	3 (2–4)
ASA‐PS ≥3	8 (80)
Clinical Frailty Scale	7 (5–7)
Active malignancy	5 (50)
Tokyo Guidelines 2018 Grade	
Grade I	4 (40)
Grade II	4 (40)
Grade III	2 (20)
Altered anatomy (Billroth‐I)	1 (10)
Rescue EUS‐GBD after failed ETGBD	1 (10)

Values are presented as median (range) or *n* (%).

ASA‐PS, American Society of Anesthesiologists Physical Status; BMI, body mass index; ETGBD, endoscopic transpapillary gallbladder drainage; EUS‐GBD, endoscopic ultrasound‐guided gallbladder drainage.

**TABLE 2:** Individual case details.

Case	Age	Sex	TG18	CCI	CFS	PT (min)	TPT (min)	F/U (mo)	Remarks
1	85	F	I	7	7	3.0	19	7	LAMS→PS
2	93	F	II	7	7	3.0	36	6	LAMS→PS
3	78	F	II	7	7	4.0	15	6	CS (EHL)
4	91	F	II	8	7	2.2	22	5	—
5	89	F	I	8	7	4.0	17	5	CS, LAMS→PS
6	77	F	I	9	7	5.0	13	4	CS (Ho: YAG)
7	98	M	III	9	7	3.0	14	—	Death POD1^†^
8	96	M	III	8	7	4.0	13	3	—
9	97	M	II	9	7	5.0	15	2.5	B‐I, rescue^‡^
10	87	M	I	7	5	3.0	23	2	—

B‐I, Billroth‐I reconstruction; CCI, Charlson Comorbidity Index; CFS, Clinical Frailty Scale; CS, cholecystoscopy; EHL, electrohydraulic lithotripsy; F/U, follow‐up; LAMS→PS, LAMS replaced with plastic stent; POD, postoperative day; PT, procedure time (puncture to stent deployment); TG18, Tokyo Guidelines 2018 severity grade; TPT, total procedure time (scope insertion to withdrawal); Ho: YAG, holmium: YAG laser lithotripsy.

^†^POD1 death in a patient with Grade III cholecystitis complicated by pre‐existing severe pneumonia and decompensated heart failure.

^‡^Rescue EUS‐GBD performed after failed endoscopic transpapillary gallbladder drainage in the same session.

All procedures were performed via the transduodenal approach using a 10‐mm LAMS. Technical success was achieved in all cases (100%). Clinical success was achieved in nine of 10 patients (90%).

### 3.2 Procedural Outcomes

All procedures were performed via the transduodenal approach using a 10‐mm LAMS (Table 3). Technical success was achieved in all 10 patients (100%). Median stent deployment time (gallbladder puncture to deployment) was 3.5 min (range, 2.2–5.0), and median total procedure time (scope insertion to withdrawal) was 16.0 min (range, 13–36). Variability in total time was primarily due to EUS observation and selection of the optimal puncture site rather than the stent deployment. No intraprocedural adverse events occurred.

**TABLE 3:** Procedural details and outcomes (*n* = 10).

Variable	Value
Approach	
Transduodenal	10 (100)
LAMS diameter, mm	10 (all cases)
Procedure time (puncture to deployment)^†^, min	3.5 (2.2–5.0)
Total procedure time (scope insertion to withdrawal)^‡^, min	16.0 (13–36)
Propofol dose, mg	115 (20–260)
Sedation time, min	22 (17–50)
Technical success	10 (100)
Clinical success^§^	9 (90)
Time to oral intake, days^¶^	1 (1–2)
Time to readiness for discharge, days^¶^	6 (4–12)
Duration of antibiotic treatment, days^¶^	6 (3–10)
WBC at admission, ×10^3^/µL	11.2 (6.3–27.1)
WBC at 48‐72 h, ×10^3^/µL ^¶^	6.5 (4.7–9.1)
WBC change, %^¶^	−31.8
CRP at admission, mg/dL	13.1 (2.4–28.2)
CRP at 48–72 h, mg/dL^¶^	5.9 (0.4–17.8)
CRP change, %^¶^	−54.4
Intraprocedural adverse events	0 (0)
Sedation‐related adverse events^‖^	1 (10)
Early adverse events (<30 days)	0 (0)
Hospital stay, days	10 (2–28)
30‐day mortality^#^	1 (10)
Follow‐up, months^**^	5 (2–7)
Recurrent cholecystitis	0 (0)
Cholecystoscopy performed	3 (30)
LAMS replaced with a plastic stent	3 (30)

Values are presented as median (range) or *n* (%).

CRP, C‐reactive protein; LAMS, lumen‐apposing metal stent; WBC, white blood cell count.

^†^Defined as the time from gallbladder puncture to the completion of stent deployment.

^‡^Includes EUS observation and puncture site selection.

^§^One patient died on POD1 before clinical success assessment and was counted as not achieving clinical success.

^¶^ *n* = 9 (One patient died on POD1).

^‖^Transient oxygen desaturation resolved with increased supplemental oxygen.

^#^POD1 death in a patient with pre‐existing severe pneumonia and heart failure.

**Nine patients with ≥1 month follow‐up.

### 3.3 Sedation‐Related Outcomes

The median propofol dose was 115 mg (range, 20–260), and the median sedation time was 22 min (range, 17–50). One patient (Case 10) experienced transient oxygen desaturation (SpO_2_ in the 80s) during the procedure, which resolved promptly with increased supplemental oxygen. No other sedation‐related adverse events were observed.

### 3.4 Clinical Outcomes and Adverse Events

Clinical improvement was achieved in of the 10 patients (90%) (Table 3). One patient (Patient 7) died on postoperative day (POD) 1 before clinical success could be assessed; this case was counted as not achieving clinical success. Inflammatory markers improved at 48–72 h post‐procedure, with median WBC and CRP reductions of 31.8% and 54.4%, respectively (*p* = 0.002 and *p* = 0.008, Wilcoxon signed‐rank test, *n* = 9; limited by small sample size). Median time to symptom resolution was 1 day (range, 1–1) in nine evaluable patients. Median time to oral intake was 1 day (range, 1–2). Median time to readiness for discharge, defined as the day when patients no longer required acute medical care, was 6 days (range, 4–12). Median antibiotic duration was 6 days (range, 3–10). No early procedure‐related adverse events (within 30 days) occurred.

One patient (Patient 7), a 98‐year‐old man with Grade III acute cholecystitis complicated by pre‐existing severe pneumonia and decompensated heart failure, died on POD1 due to septic shock and heart failure, yielding a 30‐day mortality rate of 10% (1/10). At the onset of acute cholecystitis, norepinephrine had already been initiated, and PTGBD was considered but deemed difficult because of a wandering gallbladder. This death is unlikely to be directly related to EUS‐GBD, although the contribution of procedural stress cannot be excluded.

The median hospital stay was 10 days (range 2–28). In this extremely elderly population, hospital stay was often prolonged because of transfer to nursing or long‐term care facilities rather than ongoing medical necessity.

### 3.5 Follow‐up and Additional Procedures

Among the nine remaining patients, the median follow‐up was 5 months (range, 2–7), with no recurrent cholecystitis or late stent‐related complications. Subsequent cholecystoscopy through a 10‐mm LAMS was successful in three patients using a slim upper endoscope (GIF‐1200N; outer diameter, 5.4 mm). Gallstone management included electrohydraulic lithotripsy in Patient 3 [13], spontaneous stone passage in Patient 5, and holmium: YAG laser lithotripsy in Patient 6, with complete stone clearance in all three. The LAMS was electively replaced with a plastic stent in Patients 1, 2, and 5 to prevent buried LAMS syndrome. No complications occurred during stent exchange.

## 4 Discussion

This retrospective study assessed the feasibility and short‐term outcomes of EUS‐GBD with electrocautery‐enhanced LAMS in extremely elderly and frail patients after Japanese reimbursement approval for the Hot AXIOS system. EUS‐GBD was technically successful in all patients, with clinical improvement observed in nine of 10; however, one patient with severe systemic illness died on POD1. Given the small sample size, single‐center design, and lack of a comparator group, these findings should be considered preliminary real‐world data rather than evidence of superiority or established feasibility.

To our knowledge, this is the first report focusing on EUS‐GBD outcomes in an extremely elderly population (median age, 90 years) with uniform frailty (median CFS score, 7) and a systematic frailty assessment. Previous multicenter studies have reported a mean age of 65–75 years [6, 14, 15], and frailty has rarely been evaluated. Our preliminary observations suggest that EUS‐GBD with electrocautery‐enhanced LAMS may be technically feasible in this challenging population; however, whether this translates into clinical benefit requires further investigation. The short deployment time (median, 3.5 min) may reduce procedural burden. These data may inform decision‐making in super‐aged societies.

All procedures were performed transduodenally using a 10‐mm LAMS. Recent evidence suggests fewer symptomatic adverse events with the transduodenal approach than with the transgastric approach [16]. In our cohort, median puncture‐to‐deployment and total procedure times were 3.5 and 16.0 min, respectively, indicating that most procedural time was spent on EUS assessment and puncture‐site selection. Thus, careful EUS evaluation remains essential; however, electrocautery‐enhanced LAMS enables rapid deployment once an appropriate route is identified. In extremely elderly and frail patients, internal drainage, short deployment times, and avoidance of external tubes may be advantageous.

Several endoscopic drainage options are available for high‐risk patients with acute cholecystitis. Mohan et al. [5] reported pooled technical and clinical success rates of 83% and 88% for ETGBD and 95.3% and 96.7% for EUS‐GBD, respectively. ETGBD can be limited by cystic duct tortuosity and may require prolonged cannulation and guidewire manipulation, whereas electrocautery‐enhanced LAMS permits single‐step deployment, with a reported mean deployment time of 3.1 min [17]. This may be important in extremely elderly patients, in whom prolonged sedation may increase respiratory, hemodynamic, and delirium‐related risks [18]. In our series, one patient with Billroth‐I reconstruction underwent successful EUS‐GBD after failed ETGBD, suggesting that EUS‐GBD may be a rescue option when transpapillary access is difficult. Together with previous reports in Billroth‐II anatomy, this finding supports its potential utility in selected patients with surgically altered anatomy [19, 20]. However, our findings do not imply that EUS‐GBD should replace ETGBD in all nonsurgical candidates. ETGBD may be preferable in selected settings, including antithrombotic therapy without sphincterotomy, coagulopathy or large‐volume ascites, or concomitant ERCP for choledocholithiasis [21]. Therefore, the drainage strategy should be individualized according to patient factors, anatomy, disease severity, and local expertise. EUS‐GBD with electrocautery‐enhanced LAMS may be useful when rapid internal drainage with minimal procedural complexity is required in extremely elderly and frail patients.

Although no direct comparison with PTGBD or ETGBD was performed, our technical success rate of 100% and clinical success rate of 90% (9/10) appear numerically similar to pooled EUS‐GBD success rates reported by Mohan et al. [5]; however, meaningful comparison is limited by the small sample size and lack of a control group. PTGBD remains limited by tube‐related complications [3]. Importantly, our cohort was substantially older (median age 90 vs. 65–75 years) and uniformly frail (median CFS 7), although the clinical implications remain to be determined.

Another notable finding was that subsequent cholecystoscopy was feasible through a 10‐mm LAMS using a slim upper endoscope. Although larger LAMS diameters are generally favored for trans‐stent intervention [21], selected gallbladder interventions may be feasible through a 10‐mm stent with a slim endoscope. These findings should be interpreted cautiously, but suggest the versatility of the 10‐mm LAMS beyond drainage.

The LAMS was electively replaced with a plastic stent in three patients. Although favorable long‐term outcomes after prolonged LAMS placement have been reported [22], buried LAMS syndrome and other delayed issues remain concerns [23, 24]. As an optimal long‐term strategy after EUS‐GBD has not yet been established, we adopted an individualized approach based on the patient's condition and discussions with patients and families.

The POD1 death in Patient 7 deserves attention. This patient had Grade III acute cholecystitis complicated by severe pneumonia and decompensated heart failure, and norepinephrine was initiated before acute cholecystitis was diagnosed. PTGBD was considered but deemed difficult because of a wandering gallbladder, leaving EUS‐GBD as the most feasible drainage option. Although technically successful, the patient died on POD1. This death was unlikely to be directly attributable to EUS‐GBD; however, procedural stress could not be excluded. This case suggests that EUS‐GBD may be an alternative when PTGBD is infeasible; however, successful drainage does not necessarily overcome the poor prognosis of profound systemic illness. Therefore, our findings should not be extrapolated to all patients with Grade III cholecystitis, particularly those with uncontrolled non‐biliary sepsis, and realistic expectations should be shared with patients and families.

At our institution, EUS‐GBD was considered for acute cholecystitis (Tokyo Guidelines 2018) in high surgical‐risk patients (CCI ≥6 and/or ASA‐PS ≥3), when a multidisciplinary team deemed cholecystectomy inappropriate. Careful selection is required in Grade III cases with non‐biliary sepsis, severe cardiopulmonary compromise, or hemodynamic instability requiring vasopressor support. In Case 7, propofol was minimized (20 mg), and the procedure was completed rapidly (total procedure time, 14 min) to reduce procedural stress. This case suggests that outcomes may depend strongly on the systemic illness severity.

Our study also reflects the challenges specific to healthcare delivery in a super‐aged society. The median hospital stay was 10 days, which in several cases was influenced more by transfers to nursing facilities or long‐term care hospitals than by ongoing medical needs. This underscores the importance of integrating procedural decision‐making with discharge planning and social support.

This study has several limitations. First, it is a single‐center retrospective study with a small sample size, and all procedures were performed by a single certified endoscopist, limiting the generalizability of the results. Second, the median follow‐up of 5 months is insufficient to evaluate long‐term outcomes, including recurrent cholecystitis, buried LAMS syndrome, or optimal stent exchange timing. Despite no observed late complications, long‐term prospective studies are needed to establish post‐procedural management protocols and confirm clinical durability. Third, we did not compare EUS‐GBD with PTGBD or ETGBD. Fourth, the Japanese appropriate use guidelines restrict LAMS use in gangrenous or perforated cholecystitis, which may have influenced patient selection. Finally, the POD1 death highlights uncertainty in patient selection, particularly in patients with Grade III cholecystitis complicated by severe systemic illness beyond biliary infection.

In conclusion, this pilot study describes our initial experience with EUS‐GBD using a 10‐mm electrocautery‐enhanced LAMS in extremely elderly and frail patients. Technical success was achieved in all patients, and short‐term clinical outcomes were acceptable in most; however, the POD1 death in a patient with severe systemic illness highlights the need for careful patient selection. Given the small sample size and absence of a comparator group, these preliminary findings cannot establish feasibility or safety. Larger prospective studies with appropriate comparators are warranted to define optimal indications and validate these observations.

## Author Contributions

K.K.: conceptualization, methodology, investigation, writing – original draft, and project administration. T.N., M.A., T.O., Y.S.: investigation, data curation, and resources. M.Y., D.N., N.F., H.Y., H.Ka., T.F., A.K., Y.A., H.S., M.O., and K.O.: investigation, data curation, and validation. H.Ko.: supervision, writing – review, and editing. All the authors approved the final version of the manuscript.

## Funding

The authors have nothing to report.

## Ethics Statement

This study was approved by the Institutional Review Board of the HITO Medical Center (Approval No. 20260306001) and conducted in accordance with the Declaration of Helsinki.

## Consent

Informed consent was obtained from all patients or their legal representatives.

## Conflicts of Interest

The authors declare no conflicts of interest.
